# FORESEE: Fully Outsourced secuRe gEnome Study basEd on homomorphic Encryption

**DOI:** 10.1186/1472-6947-15-S5-S5

**Published:** 2015-12-21

**Authors:** Yuchen Zhang, Wenrui Dai, Xiaoqian Jiang, Hongkai Xiong, Shuang Wang

**Affiliations:** 1 Department of Electronic Engineering, Shanghai Jiao Tong University, Shanghai 200240, China; 2 Department of Biomedical Informatics, University of California, San Diego, La Jolla, CA 92093, USA

**Keywords:** Genome-wide association study, homomorphic encryption, secure outsourcing

## Abstract

**Background:**

The increasing availability of genome data motivates massive research studies in personalized treatment and precision medicine. Public cloud services provide a flexible way to mitigate the storage and computation burden in conducting genome-wide association studies (GWAS). However, data privacy has been widely concerned when sharing the sensitive information in a cloud environment.

**Methods:**

We presented a novel framework (FORESEE: Fully Outsourced secuRe gEnome Study basEd on homomorphic Encryption) to fully outsource GWAS (i.e., chi-square statistic computation) using homomorphic encryption. The proposed framework enables secure divisions over encrypted data. We introduced two division protocols (i.e., secure errorless division and secure approximation division) with a trade-off between complexity and accuracy in computing chi-square statistics.

**Results:**

The proposed framework was evaluated for the task of chi-square statistic computation with two case-control datasets from the 2015 iDASH genome privacy protection challenge. Experimental results show that the performance of FORESEE can be significantly improved through algorithmic optimization and parallel computation. Remarkably, the secure approximation division provides significant performance gain, but without missing any significance SNPs in the chi-square association test using the aforementioned datasets.

**Conclusions:**

Unlike many existing HME based studies, in which final results need to be computed by the data owner due to the lack of the secure division operation, the proposed FORESEE framework support complete outsourcing to the cloud and output the final encrypted chi-square statistics.

## Introduction

Owing to the community effort on big data, biomedical science moves focus towards data-driven methodologies [1], which rely on collecting, integrating and analyzing large scale data. For biomedical studies, especially the genome analysis, the required storage and computational capacities may easily exceed the available resources in a single institution. Recently, cloud computing [2] emerges as a flexible alternative to support cost-effective biomedical research with big data. Researchers can rely on a cloud environment to easily scale up their studies with large scale data. However, the adopt of cloud computing in biomedical studies also yields more and more concerns about the potential data privacy risk in comparison with the local computing environment. As genome data are extremely sensitive, the storage of raw genome in a cloud may increase the disclosure risk.

The recently announced NIH policy [3] allows NIH funded studies to utilize public clouds to facilitate data analysis. However, the researchers instead of the cloud providers are responsible for the data security and privacy. Many existing attacks [4-6] also demonstrate the vulnerability of de-identified genome data. Thus, it is important to protect the privacy of genome data [7-9]. The rapid improvements of the data protection techniques make it possible to perform certain computations over encrypted data [10,11] based on homomorphic encryption.

In [12], Gentry proposed the first fully homomorphic encryption scheme to enable both addition and multiplication operations over encrypted data. Brakerski *et al*. [13,14] improved homomorphic encryption scheme based on learning with errors (LWE). Lauter *et al*. [15] presented several secure statistical algorithms for genetic association studies based on homomorphic encryption. Besides, Togan *et al*. [16] studied the integer comparison problem over homomorphic encrypted data. Recently, Graepel *et al*. [17] and Naehrig *et al*. [18] also showed that certain machine learning algorithms can be implemented using HME. Wang *et al*. [24] proposed a novel homomorphic encryption based framework to securely computing on exact logistic regression. Cheon *et al*. [19] developed a protocol for HME-based edit distance calculation that employed the greedy algorithm to obtain the upper bound of exact edit distance. Zhang *et al*. [25] improved homomorphic edit distance computation by combining path-finding algorithm and integer comparison.

In this paper, we propose the FORESEE framework to achieve secured and fully outsourced chi-square statistics computation in a public cloud. We assume that the cloud faithfully follows the protocol but may be curious of information from the received data, which is the so-called semi-honest adversary model [20]. The proposed FORESEE framework enables secure division operation over the homomorphic encrypted data and allows the cloud to directly release the study results. To be concrete, the contribution of this paper is two-fold.

• We develop a secure errorless division protocol, where a one-to-one mapping function is constructed for the floating numbers in computation and the study results can be accurately decrypted with a lookup table.

• We present a secure approximation division protocol to balance the complexity and accuracy with well-designed secure integer division in secure computation. In implementation, binary tree product and group-based computation are adopted to reduce circuit depth and the number of homomorphic multiplications.

For validation, experimental results show that the proposed FORESEE framework can identify all the significant SNPs based on the chi-square statistics with a moderate complexity using multiple slots for parallel computation.

## Method

For clarity, in the rest of this paper, we use bold symbols to represent vector and matrix variables and normal symbols for scalar variables. Without specification, $\overset{⌢}{\Delta }$ is reserved for the encrypted version of variable or function Δ and log (·) stands for the logarithm with base 2.

### Secure outsourcing GWAS

In this paper, we focus on the task of secure outsourcing GWAS in the 2015 iDASH challenge [21]. Given the genotypes from two groups over a number of single nucleotide polymorphisms (SNPs), we aim to securely calculate the chi-square statistics for the SNPs between the given case-control groups. The chi-square statistic) *χ*^2 ^is used by chi-square test to statistically assess whether there is significant association between the genetic variants and disease status. Typically, *χ*^2 ^is obtained by cumulating the normalized squared deviations between the observed and expected frequency distribution of alleles.

(1) $$\chi^{2}=\underset{i}{\sum }\underset{j}{\sum }\frac{{\left(O_{i,j}-E_{i,j}\right)}^{2}}{E_{i,j}}$$

Here, *O_i,j _*and *E_i,j _*are the observed and expected allele counts for allele *j*, e.g. *j *= 1 for allele 'A' and *j *= 2 for allele 'a' in (see Table 1) from the case (*i *= 1) or control (*i *= 2) group, respectively.

**Table 1:** Observed allele counts for SNP, where *O*_1,1 _and *O*_1,2 _are the number of alleles A and a in the case group, *O*_2,1 _and *O*_2,1 _are the corresponding counts in the control group, *N*_1 _and *N*_2 _are the total allele counts for the case and control group, respectively.

SNP	A	a	Total
Case	*O*_1,1_	*O*_1,2_	*N*_l _= *O*_1,1 _+ *O*_1,2_

Control	*O*_2,1_	*O*_2,2_	*N*_2 _= *O*_2,1 _+ *O*_2,2_

Total	*O*_1,1 _+ *O*_2,1_	*O*_1,2 _+ *O*_2,2_	*N*_l _+ *N*_l2_

Let us denote $N_{1}=O_{1,1}+O_{1,2}$ and $N_{2}=O_{2,1}+O_{2,2}$ the total number of alleles in the case and control groups, respectively. In general, *E_i,j _*is computed by $\left(\left(O_{1,j}+O_{2,j}\right)\cdot N_{i}\right)/\left(N_{1}+N_{2}\right)$ for *i *= 1, 2 and *j *= 1, 2. If we assume that the case-control groups have the same number of *n *patients, we can obtain $N_{1}=N_{2}=2n$. Thus, Equation (1) can be simplified by

(2) $$\chi^{2}=\frac{4n\cdot {\left(O_{1,1}-O_{2,1}\right)}^{2}}{\left(O_{1,1}+O_{2,1}\right)\left[4n-\left(O_{1,1}+O_{2,1}\right)\right]}$$

Equation (2) indicates that, in addition to homomorphic additions and multiplications, the χ^2 ^statistic computation over encrypted dataset requires one secure division for fully outsourced GWAS, which is not supported in many existing HME-based schemes [15,17,22]. For example, if the numerator and denominator in Equation (2) are released directly due to the lack of secure division operation, one can easily infer the underlying allele counts (i.e., *O*_1,1_and *O*_2,1_) by solving a system of equations. To address the problem, we propose the FORESEE framework to enable secure division operation for the χ^2 ^statistic computation on an untrusted cloud.

### The proposed framework

**Figure 1:**
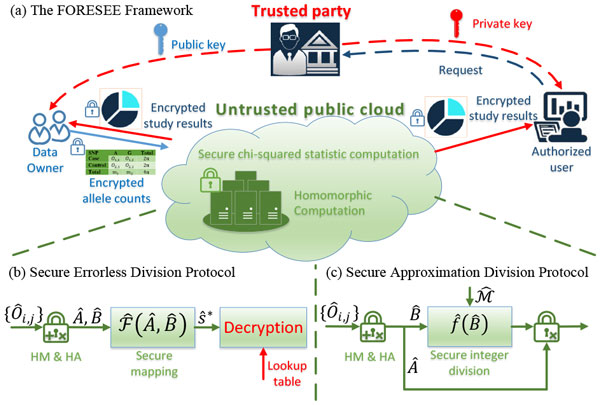
**Conceptual diagram for the proposed FORESEE framework**.

Figure 1 illustrates the proposed FORESEE framework, which allows secured and fully outsourced chi-square statistics computation in a public cloud and enable flexible release of study results. Using homomorphic encryption, the data owner can encrypt observed allele counts and directly upload to the public cloud. Consequently, the chi-square statistics can be securely computed according to Equation (2) based on homomorphic computation. Contrary to many existing HME-based schemes [15,17,22], the proposed framework develops two protocols for secure division operations over encrypted data, so that the final results are not necessarily computed by the data owner. As a result, authorized users are able to access the encrypted study results when granted the private key for decryption. Remarkably, the secrecy of uploaded sensitive information and released study results can be guaranteed under the proposed framework, as the trusted party would not interact with the untrusted public cloud. Thus, the proposed scheme enables secure outsourcing of the chi-square statistic computation to public cloud services, by which individuals or single institutions could contribute to the chi-square statistic computation in GWAS in a secure manner.

In the FORESEE framework, we develop two protocols for secure division operations, namely, secure errorless division and secure approximation division. The secure errorless protocol makes a secure one-to-one mapping from floating numbers to a set of encrypted positive integers. Consequently, authorized users can decrypt the study results with a lookup table. To achieve errorless division, the proposed protocol requires a deep circuit.

To balance the accuracy and complexity in chi-square statistic computation, the secure approximation division protocol is proposed as an alternative solution. Using secure integer division, the protocol approximates the study results with a tunable error rate. To improve its efficiency, binary tree product and group-based computation are designed to reduce circuit depth and the number of homomorphic multiplications.

In the following subsections, we will elaborate both protocols developed for the FORESEE framework.

### Secure errorless division protocol

In this section, we propose the secure errorless division protocol when both dividend and divisor are small (e.g., less than 100). Considering that secure division operation is not available in existing HME-based schemes [15,17,22], we construct a one-to-one mapping function from floating numbers to a set of encrypted positive integers. Thus, the study results can be accurately decrypted with a lookup table corresponding to the one-on-one mapping function.

#### Secure mapping for division outcomes

To map the study result (in floating numbers), we construct a function with an integer output that uniquely corresponds to the division outcomes given a dividend and divisor. Let us denote $m\in \left[0,\bar{m}\right]$ and $w\in \left[1,\bar{w}\right]$ the dividend and divisor, respectively. Here, the upper bounds $\bar{m}$ and $\bar{w}$ of *m *and *w *should be predefined, so that the lookup table for decryption can be synchronized for all the authorized users. Consequently, we construct a two-dimensional function F(m,w) that returns the positive integer *u_m,w _*corresponding to an index of the division result of *m/w *in floating number.

(3) $$F\left(m,w\right)=u_{m,w}$$

In the ciphertext domain, *u_m,w _*can be determined by the polynomials of *m *and *w *related to the ciphertext modulus *p*. According to the Fermat Theory, we can construct a simplified function with less number of homomorphic multiplications. Given the prime *p *>*mw *, the secure mapping function is

(4) $$\hat{F}\left(\hat{m},ŵ\right)\equiv \hat{m}{ŵ}^{p-2}\left(\;\mathrm{mod}\;p\right).$$

In Proposition 1, we demonstrated that the secure mapping proposed in Equation (4) is a one-to-one mapping from floating outcomes of *m*/*w *to a set of encrypted positive integers.

**Proposition 1 ***Given arbitrary positive integers m*_1_, *m*_2_, *w*_1_, *and w*_2 _*taking their values *$\left[1,|\sqrt{p}|\right]$, *they satisfy*

(5) $$\frac{m_{1}}{w_{1}}=\frac{m_{2}}{w_{2}},$$

*if and only if *$F\left(m_{1},w_{1}\right)\equiv F\left(m_{2},w_{2}\right)\left(\;\mathrm{mod}\;p\right)$*, where *$\left|\sqrt{p}\right|$*is the round function that returns the maximum integer not greater than *$\sqrt{p\;}$.

*Proof*. Please refer to Appendix I.

Proposition 1 implies that Equation (4) can map any pairs of $\left(\hat{m},ŵ\right)$ with the same irreducible fraction to the same outcome $\hat{F}\left(\hat{m}*,ŵ*\right)$, where m^* ^and w^* ^are the integer numerator and denominator, respectively that have no other common divisors. For example, given the ciphertext modulus $p=101,\hat{F}\left(\hat{2},\hat{1}\right)$ would be $\hat{2}$ for the pairs $\left(\hat{2},\hat{1}\right),\left(\hat{4},\hat{2}\right)$ and $\left(\hat{8},\hat{4}\right)$. This fact means that the encrypted outcome can be securely released, as the authorized users can only obtain the accurate irreducible fraction m^*^/w^*^, but cannot infer the exact value of $\left(\hat{m},ŵ\right)$.

Algorithm 1: Secure errorless division

0: **Inputs: **encrypted variable $\hat{m},ŵ$, upper bound $\bar{m},\bar{w}$, the ciphertext modulus *p*.

1: Let ${{ŝ}_{0}}^{*}=ŵ,{ŝ}^{*}=\hat{m}$.

2: Let $u*=\left\lfloor \text{log}\left(p-2\right)\right\rfloor$

3: Decompose *p *− 2 as $p-2=\Sigma_{i=0}^{h}2^{v_{i}}$, where h  is the number of nonzero bits in the binary representation of *p *− 2 and $v_{i}$. is the position of i-th nonzero bit.

4: **For **each *i *= 1,2,⋯, u^*^

5:   ${ŝ}_{i}^{*}={ŝ}_{i-1}^{*}*{ŝ}_{i-1}^{*}$

6: **end for**

7: **For **each *i *= 0,1,⋯,h 

8:   ${\hat{s}}^{*}={\hat{s}}^{*}*{ŝ}_{v_{i}}^{*}$

9: **end for**

10: **Outputs**: ${ŝ}^{*}$

During decryption, users can find the accurate study result  r with a lookup table, which consists of all possible irreducible fractions within ranges $m\in \left[0,\bar{m}\right]$ and $w\in \left[1,\bar{w}\right]$. Here, we provide two examples, where $\bar{m}=\bar{w}=10$ and *p *is set to 101 as the smallest prime greater than $\bar{m}\bar{w}=100$. It is worth mentioning that we can obtain the study result  r in floating number in Example 2. This fact verifies the accuracy of the proposed secure errorless division.

**Example 1 **The authorized users would obtain s^* ^= 2 by decrypting ${ŝ}^{*}=\hat{2}$. The pair of co-prime integers (*m*, *w*) corresponding to $\hat{F}\left(\hat{m},ŵ\right)\equiv \hat{2}$ (mod 101) is (2,1). Thus,  r = *m*/*w *= 2.

**Example 2 **When ${ŝ}^{*}=\hat{35},\left(m,w\right)=\left(4,3\right)$ as $\hat{F}\left(\hat{m},ŵ\right)=\hat{4}\cdot {\hat{3}}^{99}\equiv \hat{35}\left(\;\mathrm{mod}\;101\right)$. As a result,  r = 4/3.

### Secure approximation division protocol

In this subsection, we aim to develop the secure approximation division protocol. Since *n *(i.e., the number of patients in case or control group) is assumed to be a known integer, we denote *A *and *B *the dividend and divider of $\frac{{\left(O_{1,1}-O_{2,1}\right)}^{2}}{\left(O_{1,1}+O_{2,1}\right)\left[4n-\left(O_{1,1}+O_{2,1}\right)\right]}$ in Equation (2), respectively. Thus, the chi-square statistic can be rewritten as

(6) $$\chi^{2}=4n*A*\left(\frac{1}{B}\right)$$

where $A={\left(O_{1,1}-O_{2,1}\right)}^{2}$ is a nonnegative integer, and $B=\left(O_{1,1}+O_{2,1}\right)\left[4n-\left(O_{1,1}+O_{2,1}\right)\right]$ is a positive integer. Thus, given encrypted counts ${\hat{O}}_{1,1}$ and ${\hat{O}}_{2,1}$, $\hat{A}$ and $\hat{B}$ can be obtained with homomorphic multiplications and additions. Since the fraction team 1/*B*, with the value less than one, cannot be evaluated in the ciphertext domain, we scale it up by multiplying a positive integer ℳ. Therefore, the *χ*^2 ^statistic can be approximated by

(7) $$\chi^{2}=\frac{4n\cdot decrypt\left(\hat{A}\left\lfloor \frac{\hat{M}}{\hat{B}}\right\rfloor \right)}{M},$$

where $\left\lfloor M/B_{i}\right\rfloor$ is the round function that returns the maximum integer not greater than *M*/*B_i _*, e.g., $\left\lfloor 7/3\right\rfloor =2$ and $\left\lfloor 10/15\right\rfloor =0$. Here, ℳ is a public information and should be large enough, as the upper bound of relative error is determined by $1/\text{min}\left(\frac{M}{B}\right)\times 100\%=\left(\frac{400n^{2}}{M}\right)\%$.

Usually, we set $M=\text{min}\left(p-1,\left\lfloor \frac{p-1}{\text{max}\left(\frac{A}{B}\right)}\right\rfloor \right)$, where *p *is the ciphertext modulus. According to Equation (7), we develop the secure approximation division protocol based on secure integer division.

#### Secure integer division

In this subsection, we describe the secure integer division protocol to achieve secure To compute $\left\lfloor \frac{\hat{M}}{B}\right\rfloor$ o in Equation (7), we first introduce a vector ***T ***with its 6-th element defined by

(8) $$t_{i}=\left\lfloor \frac{M}{B_{i}}\right\rfloor i\in \left[1,2n\right]$$

where *B_i _*= i * (4*n*− *i*), *i *= 1,2,...,2*n *are the possible values of *B *in chi-square statistic computation (see equation (6)). Consequently, given ℳ, we define a function *f*(*x*) which satisfies

(9) $$f\left(B_{i}\right)=\left\lfloor \frac{M}{B_{i}}\right\rfloor i\in \left[1,2n\right]$$

In our implementation, a one-dimensional function is formulated using Lagrange interpolating polynomial with *x *∈ {*B*_1_,..., *B*_2*n*_}

(10) $$f\left(x\right)=\overset{2n}{\underset{i=1}{\sum }}t_{i}\frac{\prod_{\underset{l\neq i}{1\leq l\leq 2n,}}\left(x-B_{l}\right)}{\prod_{\underset{l\neq i}{1\leq l\leq 2n,}}\left(B_{i}-B_{l}\right)}$$

Since division is intractable for homomorphic encrypted data, we need to derive a surrogate function for Equation (10) that can be implemented based on homomorphic multiplications and additions. For simplicity, we denote *u_i_*. the divisor for *x *= *B_i_*. in Equation (10).

(11) $$u_{i}=\underset{1\leq l\leq 2n,l\neq i}{\prod }\left(B_{i}-B_{l}\right)$$

Consequently, we can construct a surrogate function for Equation (10) by numerically finding a set of integers *v_i_*. with 1 ≤ *v_i_*. ≤ *p *− 1 for 1 ≤ *i *≤ 2*n*, that satisfy

(12) $$u_{i}v_{i}\equiv 1\;\;\left(\;\mathrm{mod}\;\;p\right)$$

Here, *p *is the cipheretext modulus (i.e., a prime under double-CRT representation in the BGV scheme). Thus, we demonstrate the existence of {*v_i_*} in Proposition 2 to guarantee the computational tractability of *f*(*x*) in the ciphertext domain.

**Proposition 2 ***For each u_i _*= 1,2,⋯,2*n, given p *> ℳ, *at least one v_i _can be found to satisfy (12)*.

*Proof*. Please refer to Appendix II.

Substituting $1/\Pi_{1\leq l\leq 2n,l\neq i}\left(B_{i}-B_{l}\right)$ with *v_i _*in Equation (10), we can reformulate *f*(*x*) with multiplications instead.

(13) $$f\left(x\right)=\overset{2n}{\underset{i=1}{\sum }}\left[t_{i}v_{i}\underset{\underset{l\neq i}{1\leq i\leq 2n,}}{\prod }\left(x-B_{l}\right)\right]$$

We transform Equation (13) into the combination of polynomials of *x *by expanding the products and combining the coefficients.

(14) $$f\left(x\right)=\overset{2n-1}{\underset{i=0}{\sum }}{h^{\prime }}_{i}x^{i}$$

Here, ${h^{\prime }}_{i}$ is the coefficient for the *i*-th order of *x *(i.e., *x^i^*.) after polynomial expansion, which includes $v_{i}B_{l}$ and *t_i_*. In the ciphertext domain, we can construct the function $\hat{f}\left(\hat{x}\right)$ for secure integer division $\left\lfloor \hat{M}/\hat{x}\right\rfloor$.

(15) $$\hat{f}\left(\hat{x}\right)\equiv \overset{2n-1}{\underset{i=0}{\sum }}{ĥ}_{i}{\hat{x}}^{i}\left(\;\mathrm{mod}\;\;p\right)$$

where $\hat{x}\in \left\{{\hat{B}}_{1},{\hat{B}}_{2},\ldots ,{\hat{B}}_{2n}\right\}$ are finite positive encrypted integers, and ${ĥ}_{i}\in \left[\hat{0},\hat{p}-\hat{1}\right]$ is obtained by encrypting $h_{i}\equiv {h^{\prime }}_{i}\left(\;\mathrm{mod}\;\;p\right)$. We set *h_i _*= 0 with *i *> 2*n *− 1.

#### Implementation optimization

The secure integer division can be optimized to further reduce the cumulative circuit depths (CCD) and number of homomorphic multiplications (HMs). To achieve this goal, we adopt group-based computation and binary tree product to generate $\hat{f}\left(\hat{x}\right)$ in implementation.

To reduce the number of HMs, a group-based computation is adopted to calculate $\hat{f}\left(\hat{x}\right)$. The key idea of the proposed group-based optimization is to first compute a set of ${ĥ}_{c\cdot d+i}{\hat{x}}^{i}$ with *i *∈ [0, *d*], where *d *is number of elements in each group, and *c *= 0,..., *C *is the group index with the total number of groups $C=\left\lfloor \left(2n-1\right)/d\right\rfloor +1$. After grouping, we get the following equation with a reduced number of HMs.

(16) $$\hat{f}\left(\hat{x}\right)\equiv \overset{C-1}{\underset{c=0}{\sum }}\left[{\hat{x}}^{c.d}\left(\overset{d-1}{\underset{i=0}{\sum }}{ĥ}_{c\cdot d+i}{\hat{x}}^{i}\right)\right]\;\left(\;\mathrm{mod}\;\;p\right)$$

Algorithm 2 describes the generation of $\hat{X}=\left(\hat{1},\hat{x},\ldots ,{\hat{x}}^{d}\right)$ using binary tree product. The number of HMs and CCD required to calculate $\hat{X}$ can be reduced to *d *− 1 and $\left\lfloor \text{log}\left(d-1\right)\right\rfloor +1$, respectively.

**Algorithm 2: Binary tree product for generating **$\hat{X}$

0: **Inputs: **encrypted variable $\hat{x}$, the maximum power *d*

1: **For ***i *= 2,3,⋯, *d*

2:   Let $l_{1}=2^{\left\lfloor \text{log}\left(i-1\right)\right\rfloor }$.

3:   Let $l_{2}=i-l_{1}.$

4:   ${\hat{x}}^{i}={\hat{x}}^{l_{1}}\cdot {\hat{x}}^{l_{2}}.$

5: **end for**

6: **Outputs**: $\hat{X}=\left(\hat{1},\hat{x},\ldots ,{\hat{x}}^{d}\right)$

An additional optimization can be applied in equation (16) by replacing the multiplication ${ĥ}_{c\cdot d+i}{\hat{x}}^{i}$ as the summation over a total number of ${ĥ}_{c\cdot d+i}$ additions of ${\hat{x}}^{i}$ to reduce the number of HMs.

Since the time cost of HMs is larger than HAs, we determine *d *by minimizing the number of HMs. As shown in Table 9 the total number of HMs required for secure integer division is 2*C *+ *d *− 3. The number of groups *C *and the number of elements in each group *d *are selected to minimize the number of HMs $F\left(d\right)=d+2\left\lfloor \left(2n-1\right)/d\right\rfloor -3$. Given an integer $n,F\left(d\right)\approx d+\frac{2\left(2n-1\right)}{d}-3$ can obtain its minimum$2\sqrt{4n-2}-3$, only when $d=\sqrt{2\left(2n-1\right)}$. Since *d *is an integer, it is estimated by $\left\lfloor \sqrt{2\left(2n-1\right)}\right\rfloor$ to minimize *F*(*d*). Thus, *C *can be estimated by $\left\lfloor \left(2n-1\right)/d\right\rfloor +1$ accordingly. Using the optimal *d *and *C*, secure integer division can be achieved based on the encrypted function $\hat{f}\left(\hat{x}\right)$ in Equation (15). Algorithm 3 elaborates the secure integer division. In line 2, in order to obtain $\hat{X^{\prime }}$, the inputs of Algorithm 2 are set to${\hat{x}}^{d}$ and *C *− 1, respectively.

Algorithm 3: Secure integer division

0: **Inputs: **encrypted variable $\hat{x}$, group size *d *, the number of groups *C*, the ciphertext modulus *p*, the polynomial parameters *h_i_*, *i *= 0,1,...,2*n *− 1

1: Compute $\hat{X}=\left(\hat{1},\hat{x},\ldots ,{\hat{x}}^{d}\right)$ according to Algorithm 2

2: Compute $\hat{X}=\left(\hat{1},{\hat{x}}^{d},\ldots ,{\hat{x}}^{\left(C-1\right)d}\right)$ according to Algorithm 2

3: **For **each *c *= 0,1,⋯, *C *− 1

4:   **For **each *i *= 0,1,⋯, *d *− 1

5:      Calculate ${\hat{h}}_{cd+i}{\hat{x}}^{i}$

6:   **end for**

7: **end for**

8: Let $\hat{a}=\hat{0}$

9: **For **each *c *= 0,1,⋯, *C *− 1

10:   ${\hat{a}}^{\prime }=\hat{0}.$

11:   **For **each *i *= 0,1,2,⋯, *d *− 1

12:      Update ${\hat{a}}^{\prime }={\hat{a}}^{\prime }+{ĥ}_{cd+i}{\hat{x}}^{i}$

13:   **end for**

14:   Update $\hat{a}=\hat{a}+{\hat{a}}^{\prime }{\hat{x}}^{cd}$

15: **end for**

16: **Outputs**: $\hat{a}=\hat{f}\left(\hat{x}\right).$.

### Parallel computation using multiple slots

Since HME schemes with ciphertext space ${\mathbb{Z}}_{q}^{L_{s}}$ support single instruction multiple data (SIMD) with *L_s _*slots, we can use parallel computation to reduce the number of homomorphic multiplications (HMs) and homomorphic additions (HAs). Denote $\hat{a}=\left({\hat{a}}_{1},{\hat{a}}_{2},\ldots ,{\hat{a}}_{L_{s}}\right)$ and $\hat{b}=\left({\hat{b}}_{1},{\hat{b}}_{2},\ldots ,{\hat{b}}_{L_{s}}\right)$ the two encrypted ciphertexts with *L_s _*slots. SIMD is applicable to simultaneous computation of the addition $\hat{a}+\hat{b}=\left({\hat{a}}_{1}+{\hat{b}}_{1},{\hat{a}}_{2}+{\hat{b}}_{2},\ldots ,{\hat{a}}_{L_{s}}+{\hat{b}}_{L_{s}}\right)$ and $\hat{a}\cdot \hat{b}=\left({\hat{a}}_{1}\cdot {\hat{b}}_{1},{\hat{a}}_{2}\cdot {\hat{b}}_{2},\ldots ,{\hat{a}}_{L_{s}}\cdot {\hat{b}}_{L_{s}}\right)$ multiplication. In two ciphertexts, only two slots in the same position can operate with each other.

In Algorithm 1, multiple encrypted outputs can be calculated at the same time with parallel computation. When the result is returned back to the user, the user extracts the integer in each slot and search it in the lookup table. Noticeably, in the parallel computation, *m^u ^*and *n^u ^*should be selected as the upper bounds of all the dividends and divisors in the slots. Similarly, multiple slots can also be used in the secure approximation division protocol. The secure integer division developed in Algorithm 3 can be simultaneously conducted for *L_s _*pairs of inputs $\left({\hat{a}}_{i},{\hat{b}}_{i}\right),i=1,2,\ldots ,L_{s}$ using multiple slots.

## Results

In this section, we evaluate the proposed FORESEE framework, which was implemented with HElib [23], one of the most efficient open-source HME libraries based on the LWE theory [13,14]. The evaluations were made on an Ubuntu 14.04 server with Intel Xeon CPU E5-2687W @ 3.10GHz and 256 GB memory. We present the performance in the terms of time and memory cost. First, we provide the results of secure errorless division on simulated data. Moreover, we provide the performance of chi-square statistics based on the secure approximation division.

### Simulation study

Table 2 elaborates the experimental setups for the secure errorless division protocol. Given the ciphertext modulus *p*, the upper bound $\bar{m}$ (i.e., dividend) and $\bar{w}$ (i.e., divisor) is set to $\left\lfloor \sqrt{p\;}\right\rfloor$. A number of *L_s _*slots are used for parallel computation. The lifting parameter for plaintext base is set to 1. The security level is 80. The number of columns in key switching is 2. Hamming distance is 64.

**Table 2:** Experimental setups for secure errorless divisio.

$\left(\bar{m},\bar{w}\right)$	*p*	*L*	*L_s_*	Public key size	Private key size
(30,30)	907	23	678	0.67 GB	0.68 GB

(40,40)	1,601	26	1,309	1.00 GB	1.00 GB

(50,50)	2,503	29	276	0.50 GB	0.51 GB

(60,60)	3,607	30	270	0.67 GB	0.68 GB

(70,70)	4,903	31	3,144	3.30 GB	3.30 GB

(80,80)	6,421	31	342	0.81 GB	0.82 GB

(90,90)	8,101	31	309	0.81 GB	0.82 GB

(100,100)	10,007	33	5,952	3.40 GB	3.40 GB

The inputs are positive integers *m *(i.e., dividend) and *w *(i.e., divisor) with corresponding upper bounds $\bar{m}$ and $\bar{w}$. *p *is plaintext base; *L *is number of levels in modulus chain; and *L_s _*is number of slots for parallel computation. Moreover, the storage costs of key generation are also provided as reference.

Using HElib, one ciphertext can contain multiple slots to have many integers encrypted into the ciphertext with the public key. Thus, the size of the public key is related to the number of multiple slots *L_s _*in addition to the ciphertext modulus *p *and the number of levels in modulus chain *L*. Taking Table 2 for example, *L_s _*for $\left(\bar{m},\bar{w}\right)=\left(70,70\right)$ is 3144, which is greater than most ones. Thus, its ciphertext sizes are much larger than the other configurations with close values of $\bar{m}$ and $\bar{w}$.

Using HElib, we are able to evaluate all the slots in the ciphertext in parallel. Table 3 shows the average execution time for the secure errorless division protocol. Based on the lookup table generated for various parameters $\left(\bar{m},\bar{w}\right)$, the proposed protocol is efficient for secure division operation over *m *≤ 100 and *w *≤ 100. However, its circuit depths increase rapidly with the growth of *m *and *w*, which limits its application for larger dividends and divisors.

**Table 3:** Time cost in seconds for key generation, encryption, and errorless division computation using various parameters.

$\left(\bar{m},\bar{w}\right)$	Key generation	Encryption	Execution time	
			
			Total	Average
(30,30)	44.8395	9.13476	9.13476	0.0135

(40,40)	64.4364	9.61973	9.61973	0.0074

(50,50)	76.5504	11.3389	11.3389	0.0411

(60,60)	73.7685	12.0161	12.0161	0.0445

(70,70)	117.846	13.9717	13.9717	0.0044

(80,80)	87.9779	4.14286	13.3369	0.0390

(90,90)	87.9528	4.10576	15.5242	0.0502

(100,100)	127.002	16.7352	16.7352	0.0028

The average execution time is obtained by averaging over all the slots used.

### Chi-square statistic computation

We employ the secure approximation division protocol in secure chi-square statistic computation. Two datasets from iDASH genome privacy protection challenge are used for evaluation, which contain 311 SNPs and 610 SNPs, respectively in the case-control groups, each consisted of with 200 individuals,

In homomorphic encryption, the ciphertext modulus *p *and the number of levels in modulus chain *L *are set to 25600000039 and 51, respectively. The public and private key sizes are both around 2.6 GB. The lifting parameter for plaintext base is set to 1. The security level is 80. The number of columns in key switching is 2 and the Hamming distance is 64. To reduce computational complexity, we use *L_S _*= 864 slots in parallel computation. For secure integer division, ℳ is 25600000000. f and a are accordingly set to 28 and 15 for 200 individuals in each group.

Table 4 provides the time cost for homomorphic evaluation of both datasets in chi-square statistics computation, including key generation, encryption, total and average execution time. Using multiple slots, the secure approximation division protocol can achieve the chi-square statistics computation in less than one second in average. Table 5 evaluates the accuracy of computation in terms of the mean-squared error (MSE) and maximum error between the exact and the approximate chi-square statistics, where the MSE are less than 5 × 10^−10 ^. The evaluation on maximum error also supports the conclusion.

**Table 4:** Time cost in seconds for key generation, encryption, and the computation of chiRsquare statistics using different parameters based on secure approximation division.

# of SNPs	Key generation	Encryption	Execution time	
			
			Total	Average
311	212.32	355.61	286.75	0.92

610	222.53	428.05	315.67	0.52

The average execution time is obtained by averaging over all the slots used.

**Table 5:** Recall and precision with different *p*-value cutoffs in identifying significant SNPS on both datasets, where the mean-squared error (MSE) and maximum error between the exact and the approximate chi-square statistics

# of SNPs	*p-*value cutoff	# of significant SNPs	Precision	Recall	**MSE **(×10^−10^)	**Maximum error **(×10^−6^)
311	0.05	24	1	1	3.21	5.5

	0.01	20	1	1		

	0.005	20	1	1		

610	0.05	56	1	1	4.07	6.0

	0.01	27	1	1		

	0.005	23	1	1		

Remarkably, we also computed the *p*-value for each SNP based on the chi-square statistic and applied different *p*-value cutoffs as 0.05, 0.01, and 0.005. The secure approximation division protocol is demonstrated to find out all the significant SNPs for both datasets under different *p *-value cutoffs. As a result, the proposed protocol provides a good tradeoff between accuracy and complexity for secure chi-square statistic computation.

Furthermore, we compare the two proposed protocols in the chi-squared statistics computation. For secure errorless division protocol, we use the same parameters list above, except that *L *is set to 151 to guarantee the required circuit depth in implementation. Table 6 and 7 compare the computational complexity and storage cost for the two protocols, respectively. In Table 6 the secure errorless division protocol requires about 10, 20 and 5 times in complexity for key generation, encryption, and execution (computation), when compared with the secure approximation division protocol. Table 7 shows that the ciphertext key sizes for secure errorless division are about 8 times larger due to the greater *L*. These results imply that the secure approximation division protocol provides a good trade-off in terms of complexity and accuracy for chi-squared statistic computation.

**Table 6:** Time cost in seconds for key generation, encryption and the computation of chi8squared statistics using different parameters based on secure errorless division (ED) and secure approximation division (AD) protocols.

# of SNPs	Key generation		Encryption		Execution time	
	
	ED	AD	ED	AD	ED	AD
311	2206	**212.3**	9575	**355.6**	1900	**287**

610	2131	**222.5**	9026	**428.1**	1660	**316**

**Table 7:** *L *(i

# of SNPs	*L*		Public key size		Private key size	
	
	ED	AD	ED	AD	ED	AD
311	151	**51**	23.3GB	**2.6GB**	23.6GB	**2.6GB**

610						

## Discussions

In this section, we analyze the computational complexity of the proposed FORESEEE protocol and discuss its potential extension and its limitation.

### Complexity analysis

In this subsection, we make an analysis on the computational complexity of the secure errorless division and secure approximation division protocols. Cumulative circuit depth2 (CCD) and the numbers of homomorphic multiplications (HMs) are provided for both protocols in the FORESEE framework.

We begin with the complexity analysis for secure errorless division protocol (i.e., Algorithm 1). As shown in Table 8 the number of HMs to calculate ${{ŝ}_{i}}^{*}$ at each iteration in A1 line 5 is 1. The number of HMs to obtain s^* ^at each iteration in A1 lines 8 is also 1. Therefore, the CCD in calculating ${{ŝ}_{i}}^{*}$ in A1 lines 4-6 are $\left\lfloor \text{log}\left(p-2\right)\right\rfloor$. The depths to obtain s^* ^in A1 lines 7-9 are $\left\lfloor \text{log}\left(p-2\right)\right\rfloor +h+1$

**Table 8:** Complexity analysis in terms of cumulative circuit depth2 (CCD) and the number of homomorphic multiplications (HMs) for secure errorless division protocol (Algorithm 1).

Algorithm 1	CCD	# of HMs
1: Let ${ŝ}_{0}*=ŵ,ŝ*=\hat{m}$.		

2: Let $u^{*}=\left\lfloor \text{log}\left(p-2\right)\right\rfloor$		

3: Decompose $p-2=\overset{h}{\underset{i=0}{\sum }}2^{v_{i}}$	−	−

4:**For **each *i *= 1,2, ⋯, u*		

5: ${ŝ}_{i}^{*}={ŝ}_{i-1}^{*}*{ŝ}_{i-1}^{*}$	$\left\lfloor \text{log}\left(p-2\right)\right\rfloor$	1

6: **end for**		

7: **For **each i = 0,1, ⋯, h		

8: ${ŝ}^{*}={ŝ}^{*}*{ŝ}_{v_{i}}^{*}$	$\begin{array}{l} 1+h\\ +\left\lfloor \log(p-2)\right\rfloor \end{array}$	1

9: **end for**		

**Total:**	$\begin{array}{l} 1+h\\ +\left\lfloor \log(p-2)\right\rfloor \end{array}$	$\begin{array}{l} 1+h\\ +\left\lfloor \log(p-2)\right\rfloor \end{array}$

Table 9 provides the CCD and number of HMs for secure approximate division (i.e., Algorithm 3). The number of HMs to obtain $\hat{X}$ and ${\hat{X}}^{\prime }$ are *d *− 1 and *C *− 2, respectively. To evaluate Equation (18), the total number of HMs are *d *+ 2*C *− 3. By using binary tree product based optimization, the circuit depths required for computing $\hat{X}$ and ${\hat{X}}^{\prime }$ are $\left\lfloor \text{log}\left(d-1\right)\right\rfloor +1$ and $\left\lfloor \text{log}\left(c-2\right)\right\rfloor +1$,respectively. Finally, the total CCD for secure approximate division operation is $\left\lfloor \text{log}\left(c-2\right)\right\rfloor +\left\lfloor \text{log}\left(d-1\right)\right\rfloor +3$.

**Table 9:** Complexity analysis in terms of cumulative circuit depth2 (CCD) and the number of homomorphic multiplications (HMs) for secure approximation division (Algorithm 3).

Algorithm 3	CCD	# of HMs
1: Compute $\hat{X}$	$\left\lfloor \text{log}\left(d-1\right)\right\rfloor +1$	*d *− 1

2: Compute ${\hat{X}}^{\prime }$	$\left\lfloor \text{log}\left(C-2\right)\right\rfloor +\left\lfloor \text{log}\left(d-1\right)\right\rfloor +2$	*C *− 2

3:**For ***c *= 0,1, ⋯, *C *− 1		

4: **For ***i *= 0,1, ⋯, *d *− 1		

5: Calculate ${\hat{h}}_{cd+i}{\hat{x}}^{i}$	−	−

6: **end for**		

7: **end for**		

**8: **$\hat{a}=\hat{0}$		

9: **For ***c *= 0,1, ⋯, *C *− 1		

10: ${\hat{a}}^{\prime }=\hat{0}$	−	−

11: **For ***i *= 0,1, ⋯, *d *− 1		

12: ${\hat{a}}^{\prime }={\hat{a}}^{\prime }+{ĥ}_{cd+i}{\hat{x}}^{i}$	−	−

13: **end for**		

14: $\hat{a}=\hat{a}+{\hat{a}}^{\prime }{\hat{x}}^{cd}$	$\left\lfloor \text{log}\left(C-2\right)\right\rfloor +\left\lfloor \text{log}\left(d-1\right)\right\rfloor +3$	1

15: **end for**		

**Total:**	$\left\lfloor \text{log}\left(C-2\right)\right\rfloor +\left\lfloor \text{log}\left(d-1\right)\right\rfloor +3$	2*C *+ *d *− 3

### Potential extension

In this paper, we proposed the FORESEE framework to address the problem of fully outsourcing chi-square statistic computation to a public cloud. However, the application scenarios for the FORESEE framework, especially the secure approximation division protocol, can be further extended to securely compute other statistics tests that involve division operations. One intuitive example is the Transmission disequilibrium test (TDT) developed to assess the genetic linkage between the genetic variants and disease status in family-based association studies. TDT is based on the binomial test with one degree of freedom, which is asymptotically equivalent to the chi-square hypothesis test.

### Limitation

There are several limitations in the FORESEE framework. First, in secure approximation division protocol, the upper bound of approximation error depends on the ciphertext modulus G. Therefore, G should be large enough to guarantee the accuracy in computation, which degrades the efficiency of the FORESEE framework. Second, the computational and storage costs based on homomorphic encryption are still very high. For example, key generation and encryption is much more timeconsuming than computation. The ciphertext sizes are also a heavy burden for communication. Finally, it is still a challenging problem to generalize the secure errorless division protocol. In summary, there is still room to improve the proposed division protocols in the FORESEE framework through better algorithm design, efficient coding in the HElib and parallelization.

## Conclusion

In this paper, we proposed a novel FORESEE framework for the secure outsourcing GWAS in the iDASH genome privacy protect challenge, especially for the chi-square statistic computation. The proposed framework consists of two protocols for secure division operation, namely secure errorless division and secure approximation division. The secure errorless protocol made a bijection between floating numbers and a set of encrypted positive integers. Thus, it could output the accurate study results based on a lookup table. On the other hand, the secure approximation division protocol adopted secure integer division to obtain approximate study results with a tunable accuracy. The protocol was able to balance the complexity and accuracy by using the group-based computation and binary tree product with improved efficiency. In comparison to existing HME-based schemes [15,17,22], both protocols enabled fully outsourced secure GWAS in an untrusted public cloud and could directly release study results to authorized users for decryption. Experimental results show that the secure approximation division protocol can capture all the significant SNPs in chi-square statistic computation with a moderate computational complexity.

## Appendix I: Proof of Proposition 1

Since $m_{1}w_{1}^{p-2}\equiv m_{2}w_{2}^{p-2}\left(\;\mathrm{mod}\;\;p\right)$, we can obtain Equation (17) by multiplying *w*_1_*w*_2 _on the both sides.

(17) $$w_{2}m_{1}w_{1}^{p-2}\equiv w_{1}m_{2}w_{2}^{p-2}\left(\;\mathrm{mod}\;\;p\right)$$

According to the Fermat's little theorem,

(18) $$w_{i}^{p-1}\equiv 1\left(\;\mathrm{mod}\;\;p\right)\;i=1,2.$$

When *w*_1 _and *w*_2 _are coprime with *p*, we can find that

(19) $$w_{2}m_{1}\equiv w_{1}m\left(\;\mathrm{mod}\;\;p\right)$$

Since $w_{2}m_{1}\leq \left\lfloor \sqrt{p}\right\rfloor *\left\lfloor \sqrt{p}\right\rfloor <p$ and $w_{1}m_{2}\leq \left\lfloor \sqrt{p}\right\rfloor *\left\lfloor \sqrt{p}\right\rfloor <p$, it holds for the prime *p *that

(20) $$-p<w_{2}m_{1}-w_{1}m_{2}<p$$

From Equations (19) and (20), we obtain that $w_{2}m_{1}=w_{1}m_{2}$, which comes to Proposition 1.

## Appendix II: Proof of Proposition 2

We recall the Fermat's little theorem that, given a prime *p*,

(21) $$q^{p-1}\equiv 1\left(\;\mathrm{mod}\;\;\;p\right)$$

where *p *and *q *are coprime numbers. Since $u_{i}\in \left[1,p-1\right]$, the greatest common divisor for *u_i _*and *p *is always 1. Given an integer *u_i _*, we can derive ${v^{\prime }}_{i}=u_{i}^{p-2}$ to satisfy $u_{i}{v^{\prime }}_{i}\equiv 1$ (mod *p*) in Equation (12). Thus, by considering $v_{i}\equiv {v^{\prime }}_{i}$ (mod *p*), *v_i_*. ∈ [1, *p *− 1] can be found for Equation (12). As a result, we draw the Proposition 2.

## Abbreviations

GWAS: GenomeRwide association study; HME: Homomorphic Encryption; HM: Homomorphic Multiplication; CCD: Cumulative circuit depth.

## Competing interests

The authors declare that they have no competing interests.

## Authors' contributions

YZ and WD drafted the majority of the manuscript, YZ conducted the experiments. HX and XJ provided some helpful comments. SW provided the motivation for this work, detailed edits and critical suggestions.

## References

[B1] Howe D Costanzo M Fey P Gojobori T Hannick L Hide W Hill DP Kania R Schaeffer M St Pierre S Twigger S White O Rhee SY Big data: The future of biocuration Nature 2008 455 47 50 18769432 10.1038/455047a PMC2819144

[B2] The NIST Definition of Cloud Computing National Institute of Standards and Technology

[B3] NOTLODL15L086: Notice for Use of Cloud Computing Services for Storage and Analysis of ControlledLAccess Data Subject to the NIH Genomic Data Sharing (GDS) Policy http://grants.nih.gov/grants/guide/noticeRfiles/NOTRODR15-086.html

[B4] Homer N Szelinger S Redman M Duggan D Tembe W Muehling J Pearson JV Stephan DA Nelson SF Craig DW Resolving individuals contributing trace amounts of DNA to highly complex mixtures using highLdensity SNP genotyping microarrays PLoS Genet 2008 4 e1000167 18769715 10.1371/journal.pgen.1000167 PMC2516199

[B5] Gymrek M McGuire AL Golan D Halperin E Erlich Y Identifying personal genomes by surname inference Science (80-) 2013 339 321 324 10.1126/science.1229566 23329047

[B6] Wang R Li YF Wang X Tang H Zhou X Learning your identity and disease from research papers Proceedings of the 16th ACM conference on Computer and communications security - CCS '09 2009 New York, New York, USA: ACM Press 534 44

[B7] Naveed M Ayday E Clayton EW Fellay J Gunter CA Hubaux JRP Malin BA Wang X Privacy and Security in the Genomic Era 2014 10.1145/2767007 PMC4666540 26640318

[B8] Wang S Mohammed N Chen R Differentially private genome data dissemination through topLdown specialization BMC Med Inform Decis Mak 2014 14 Suppl 1 S2 25521306 10.1186/1472-6947-14-S1-S2 PMC4290801

[B9] Kamm L Bogdanov D Laur S Vilo J A new way to protect privacy in largeLscale genomeLwide association studies Bioinformatics 2013 29 886 93 23413435 10.1093/bioinformatics/btt066 PMC3605601

[B10] Zhou M Zhang R Xie W Qian W Zhou A Security and Privacy in Cloud Computing: A Survey 2010 Sixth International Conference on Semantics, Knowledge and Grids. IEEE 2010 105 112

[B11] Wang W Hu Y Chen L Accelerating fully homomorphic encryption using GPU IEEE Conference on High Performance Extreme Computing (HPEC) 2012 1 5

[B12] Gentry C Fully homomorphic encryption using ideal lattices Proceedings of the 41st annual ACM symposium on Symposium on theory of computing - STOC '09 2009 New York, NY, USA: ACM Press 169 178

[B13] Brakerski Z Gentry C Vaikuntanathan V (Leveled) fully homomorphic encryption without bootstrapping Proceedings of the 3rd Innovations in Theoretical Computer Science Conference on - ITCS '12 2012 111 New York, NY, USA: ACM Press 309 325

[B14] Brakerski Z Vaikuntanathan V Efficient fully homomorphic encryption from (standard) LWE SIAM J Comput 2011 43 831 871

[B15] Lauter K LópezRAlt A Naehrig M Private computation on encrypted genomic data14th Privacy Enhancing Technologies Symposium, Workshop on Genome Privacy (GenoPri'14) 2014 Amsterdam, The Netherlands

[B16] Togan M Plesca C ComparisonLbased computations over fully homomorphic encrypted data Communications (COMM), 2014 10th International Conference on 2014 1 6

[B17] Graepel T Lauter K Naehrig M ML confidential: Machine learning on encrypted data Information Security and Cryptology ICISC 2012 2013 Springer 1 21

[B18] Naehrig M Lauter K Vaikuntanathan V Can homomorphic encryption be practical? Proceedings of the 3rd ACM workshop on Cloud computing security workshop - CCSW '11 2011 New York, NY, USA: ACM Press 113

[B19] Cheon JH Kim M Lauter K Homomorphic Computation of Edit Distance WAHC'15 - 3rd Workshop on Encrypted Computing and Applied Homomorphic Cryptography 2015

[B20] Hazay C Lindell Y Efficient Secure Two-Party Protocols 2010 Berlin, Heidelberg: Springer Berlin Heidelberg Information Security and Cryptography

[B21] 2015 iDASH Privacy and security Workshop http://www.humangenomeprivacy.org/2015/

[B22] Bos JW Lauter K Naehrig M Private predictive analysis on encrypted medical data J Biomed Inform 2014 50 234 243 24835616 10.1016/j.jbi.2014.04.003

[B23] https://github.com/shaih/HElib

[B24] Wang S Zhang Y Dai W Lauter K Kim M Tang Y Xiong H Jiang X HEALER: Homomorphic computation of ExAct Logistic rEgRession for secure rare disease variants analysis in GWAS Bioinformatics 2015 [accepted] 10.1093/bioinformatics/btv563 PMC4739182 26446135

[B25] Zhang Y Dai W Wang S Kim M Lauter K Sakuma J Xiong H Jiang X SECRET: Secure EditLdistance computation over homomoRphic Encrypted daTa Proceedings of the 5th Annual Translational Bioinformatics Conference Tokyo, Japan 2015 [accepted]

